# Discordant population histories of host and its parasite: A role for ecological permeability of extreme environment?

**DOI:** 10.1371/journal.pone.0175286

**Published:** 2017-04-10

**Authors:** Dagmar Jirsová, Jan Štefka, Miloslav Jirků

**Affiliations:** 1 Faculty of Science, University of South Bohemia, Branišovská, České Budějovice, Czech Republic; 2 Institute of Parasitology, Biology Centre, Czech Academy of Sciences, Branišovská, České Budějovice, Czech Republic; University of Pretoria, SOUTH AFRICA

## Abstract

Biogeographical and ecological barriers strongly affect the course of micro-evolutionary processes in free living organisms. Here we assess the impact of a recently emerged barrier on populations of limnic fauna. Genetic diversity and population structure in a host-parasite system (*Wenyonia virilis* tapeworm, *Synodontis schall* catfish) are analyzed in the recently divided Turkana and Nile basins. The two basins, were repeatedly connected during the Holocene wet/dry climatic oscillations, following late Pleistocene dessication of the Turkana basin. Mitochondrial DNA sequences for *cytochrome oxidase I* gene (*cox I*) and a whole genome scanning method—amplified fragment length polymorphism (AFLP) were employed. A total of 347 *cox I* sequences (representing 209 haplotypes) and 716 AFLP fragments, as well as 120 *cox I* sequences (20 haplotypes) and 532 AFLP fragments were obtained from parasites and hosts, respectively. Although results indicate that host and parasite populations share some formative traits (bottlenecks, Nilotic origin), their population histories/patterns differ markedly. Mitochondrial analysis revealed that parasite populations evolve significantly faster and show remarkably higher genetic variability. Analyses of both markers confirmed that the parasites undergo lineage fission, forming new clusters specific for either freshwater or saline parts of Lake Turkana. In congruence with the geological history, these clusters apparently indicate multiple colonisations of Lake Turkana from the Nile. In contrast, the host population pattern indicates fusion of different colonisation waves. Although fish host populations remain connected, saline habitats in Lake Turkana (absent in the Nile), apparently pose a barrier to the gene flow in the parasite, possibly due to its multihost lifecycle, which involves freshwater annelids. Despite partially corroborating mitochondrial results, AFLP data was not sufficiently informative for analyzing populations with recently mixed biogeographic histories.

## Introduction

The cyclic Pleistocene climatic oscillations severely affected all biomes. Alternating hot-humid and cool-dry periods lasting for tens of millennia caused repeated contraction/expansion and/or fragmentation of vegetational belts and hydrological systems [1]. Freshwater habitats and taxa were among the most severely affected [2–4]. Contractions/fragmentations of hydrological systems during the cold-dry periods resulted in patchy distribution of limnic biota, which remained restricted to areas with continuous freshwater habitats. In the tropics such humid refugia allowed localized persistence and accumulation of both aquatic and terrestrial taxa during the critical cold-dry periods and these are recognized as biodiversity and/or endemism centres analogous to the Pleistocene refugia of the Holarctic [4,5]. During the cold-dry periods, desiccation, shrinkage and fragmentation affected all major drainages and lakes worldwide, making some of them isolated and endorheic and consequently, in arid areas, subject to salinization. Yet, the significance of African Pleistocene refugia for speciation processes remains unclear, since many of the radiations associated with them are of Miocene or older ages (African vertebrates: phylogenetic evidence e.g. [6–8]; indirect paleontological evidence e.g. [9,10]) and because there is only limited information available on millennial-scale genetic processes in metazoans [11,12].

Lundberg and colleagues [13], further supported by [14], proposed a ‘hydrogeological hypothesis’ based on the finding that Neotropical fish diversity might be a result of geologically and climatically controlled palaeohydrological changes. Recently, similar relationships between phylogenetic history and hydrogeological changes have been revealed in two Afrotropical fish genera, an alestid *Hydrocynus* and a mochokid *Synodontis* [8,15,16]. The latter two studies highlighted the significance of two geological structures, the Central African shear zone and the East African rift system, as the drivers of diversification and subsequent allopatric speciation.

Mechanisms of allopatric speciation by geology- and climate-driven isolation/fragmentation of drainages are well explained. On the other hand, the effects of isolation-related hydrological changes on limnic biotas could be more complex. Despite the obvious complementarity of lake isolation and salinization observed in numerous contemporary lakes in arid regions (in Africa for example lakes Chad, Natron and Turkana), the significance of salinity itself for the intraspecific diversification and/or speciation remains virtually unknown. As a result, allopatric speciation mostly serves as a universal explanation of diversity and biogeography of African freshwater organisms. One of the few exceptions is the well-known phenomenon of sympatric speciation in African cichlids [12,17].

The present study provides an analysis of evolutionary processes on a millennial scale, 5 thousand years ago (kya) following the Holocene split of initially continual populations of fish and their parasites in geo-historically and biogeographically allied Nile and Turkana basins, following the Late Pleistocene (near) dessication of Lake Turkana and its reestablishment and expansion during the African humid period [18,19]. The main aim of the study is to show that metazoan evolutionary processes can be detected within short time periods (millennia) on a population genetic level [20,21]. Based on this evidence we predict to see an emerging population structure between the two recently divided units (Nile and Turkana). In addition, we test the hypothesis, that apart from the well-understood effects of isolation (by fragmentation, distance etc.), salinization of limnic systems also poses a significant evolutionary force that can shape population structure on short spatial scales and might facilitate diversification in otherwise strictly freshwater organisms [22]. These hypotheses are assessed by testing two assumptions: a) the Nile represents a source/ancestral biogeographic unit from which Lake Turkana is derived, and b) there is a population genetic signal mirroring a recent (African humid period ~11–5 kya) establishment and subsequent five millennia of isolation of extant biota of Lake Turkana, and/or its marked environmental gradient of salinity. In order to deepen the knowledge on the degree of genetic changes taking place on millennial time scales, this study addresses the following partial aims: i) evaluation of intra- and inter-population genetic variability of hosts and parasites within and between the Nile and Turkana basins and assessment of factors potentially responsible for the observed patterns, e.g. selection pressures, bottlenecks, salinity; (ii) evaluation, whether or not the parasite and/or host population genetic patterns indicate on-going evolutionary processes potentially leading to speciation; (iii) comparison of the rate of evolution between host and parasite in connection with differences in the depth of population structure.

## Methods

### Geographic model

We proposed the following geographic model consisting of two (bio)geographic sub-systems: a non-isolated and fully freshwater basin, and an isolated endorheic basin with marked salinity gradient. Our criteria were specified for the two geographic sub-systems: i) absence of aquatic link; ii) model organisms common in both subsystems; iii) clinal salinity gradient present in the isolated sub-system; iv) isolated sub-system of sufficient size to omit size-dependent bias; v) recent disconnection of the two sub-systems.

**Fig 1:**
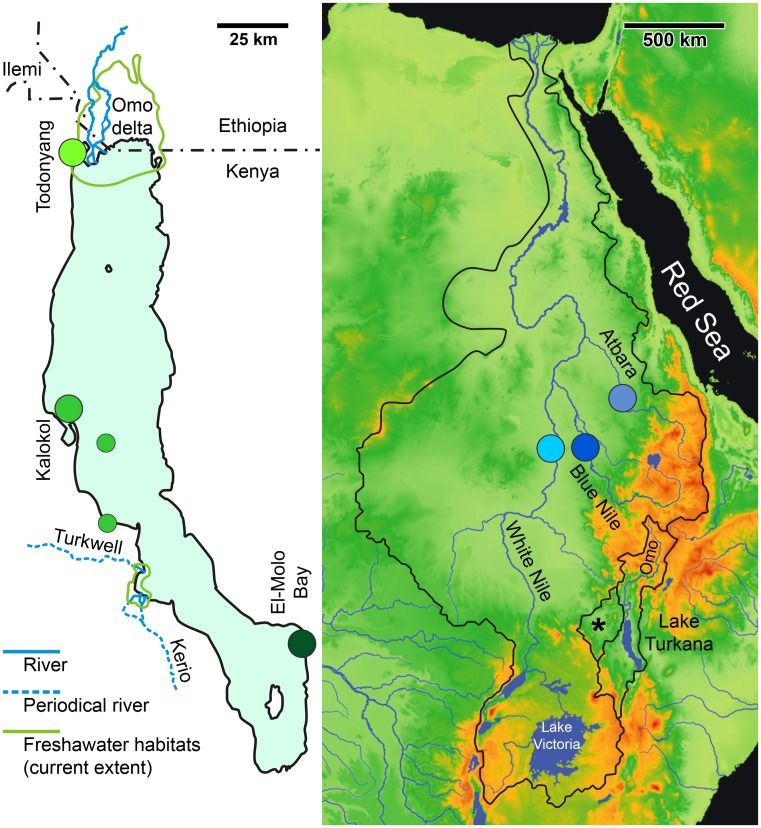
Maps of Lake Turkana (left) and the Nile basin (right) showing the locations of our sampling localities and the outlines of the separate basins (based on [28,29] and original observations). The asterisk indicates the current endorheic Lotikipi Plain, which is being temporarily flooded during exceptionally rainy years. During the wet Pleistocene climatic phases and the African humid period of the Holocene, the Lotikipi Plain repeatedly posed an aquatic link between the Nile and Turkana basins.

Approximately 12.5 kya, the Victoria Nile, an endorheic lake until then, and the White Nile system merged and became part of the main Nile system to form its contemporary extent [23]. Lake Turkana is the largest desert lake on Earth measuring ~240 by 13–50 km, with surface area 6,750 km^2^, volume 203 km^3^ and average/max. depth 31/114 m. In the Late Pleistocene 35–15 kya, Lake Turkana almost completely dried out. During the Holocene, the peak of the African humid period was 11.5–10.5 kya, followed by brief periods (9.0, 6.6 and 5.2 kya), when the lake spilled over the Lotikipi Plain to the north-west, overflowed into White Nile [19] and became a part of Nile-Chad-Niger limnic system (Fig 1). During the drier, lower level periods, similar to the present, the Nile-Turkana link disappeared and the Turkana basin became endorheic [24–26]. The near desiccation of Lake Turkana during the Late Pleistocene, followed by temporary connection with the Nile is the reason for the recent origin of its limnic biota, the faunistic similarity to the Nile and relatively low levels of endemism (~60 fish spp. including 10 endemics, [27]). Currently, the arid area separating the Nile and Turkana basins lacks permanent aquatic environments.

There is no outlet and with reduced inflows and high evaporation the chloro-carbonate alkaline water is subject to significant water level fluctuations (1–3 m annually, > 20 m during 20th century). The lake is becoming increasingly saline along the Omo River delta (0.07) in the north, to highly saline (brackish) towards the south (Kalokol 1.78, El-Molo 1.89) (original unpublished data, values obtained after the peak of rainy season 2–26 September 2008). The Omo delta harbours rich Nilotic biota, whereas the major saline part of Turkana possesses depleted communities [27].

### Model organisms

Model organisms were selected according to their wide distribution, high abundance and reliable availability in both basins and at all localities. Additional criteria for the parasite model included narrow host specificity, complex life cycles (more likely to be influenced by salinity) and well resolved taxonomy.

The endemic African catfish genus *Synodontis* Cuvier (Siluriformes: Mochokidae) comprises ~120 nominal species [30]. Most species are freshwater, benthopelagic, potamodromous and omnivorous, feeding on detritus, algae, plants, invertebrates, small fish and carrion. The life history is not well known, albeit *Synodontis* spp. can mature sexually before one year of age (M. Jirků & R. Blazek, pers. obs.). *Synodontis schall*, the only representative of the genus in Lake Turkana, was pre-selected as a model host due to its high abundance in both basins and association with several host-specific parasite taxa. *Synodontis nigrita* was selected as an outgroup due to its clear distinctiveness from its congeners and its availability in the study area.

The endemic African tapeworm genus *Wenyonia* Woodland, 1923 (Caryophyllidea: Caryophyllaeidae) comprises six species exclusively parasitizing *Synodontis* hosts. Our model, *Wenyonia virilis* Woodland, 1923, is the most widespread and abundant representative and unlike its congeners, present in both basins [31]. The life cycle of *Wenyonia* is unknown but probably similar to other fish caryophyllideans, i.e. larval development in freshwater oligochaetes, no second intermediate host, maturation in the fish definitive host. In the related genus *Khawia*, maturation in the fish takes 1.5–6 months [32,33]. Adult longevity is also unknown but probably less than a year.

### Specimen collection and DNA extraction

Parasites and fish hosts were collected from Lake Turkana and the Nile at eight localities (Table 1). The Turkana localities were chosen to cover the salinity gradient from freshwater to saline environments. Fish examined were obtained in fish markets and/or from fishermen under supervision of Kenya Marine and Fisheries Research Institute authorities. Since fish were destined for human consumption, no permission was needed to collect these fish. Fish that were still alive when examined were killed by dorsal pithing (spinal cord and blood vessels cut immediately behind the head), a method compliant with Kenyan, Czech and European legislation. All fish were euthanized and processed for dissection shortly after purchase, no further manipulation was involved.

**Table 1:** Sampling localities, coordinates and sample sizes.

Locality	Coordinates	Wenyonia	Synodontis
Lake Turkana			
El-Molo Bay—saline	2.832222 N, 36.695833 E	110	30
Kalokol—medium salinity	3.558611 N, 35.915833 E	1	4
Kerio River delta—medium salinity	2.974813 N, 36.173469 E	0	6
Central Island—medium salinity	3.495833 N, 36.040278 E	0	1
Todonyang, Omo delta—feshwater	4.451667 N, 35.94388 E	170	50
Nile River			
White Nile in Kostí	13.172222 N, 32.672222 E	75	26
Blue Nile in Sennar	13.543611 N, 33.636667 E	7	21
Atbarah River in Khashm el-Girba	14.919444 N, 35.901944 E	5	0
Totals (n)		368	138

A small piece of tissue from the worm and host liver was cut off, washed extensively in physiological saline solution and stored in 96% ethanol. The remaining part of each cestode was fixed with hot 4% buffered formalin and stained with Schuberg’s hydrochloric carmine solution [34]. Vouchers were deposited in the helminthological collection of the Institute of Parasitology BC-CAS, České Budějovice, Czech Republic (*W*. *virilis*, IPCAS C-503; *W*. *minuta*, IPCAS C-571; *W*. *youdeoweii*, IPCAS C-573). Genomic DNA was extracted using a standard phenol chloroform extraction method [35].

### PCR

Specific primers, Wen-cox F3 (5’ AGAGAGCGGTTACTGCTAATAA 3’) and Wen-cox R3 (5’ ATAATGAAAGTGCGCTACTACAAATCA 3’), were designed using sequences of related taxa from GenBank for amplifying and sequencing a partial region (~ 1100 bp) of cytochrome oxidase I gene (*cox I*) in *Wenyonia*. A PPP Master Mix (Top Bio) was used to prepare PCR reactions containing 12.5 μl of the PPP Master mix, 10 μl of ultra pure water, 5 pM of each PCR primer and 1.5 μl of isolated DNA (50 to 150ng). Cycling conditions were as follows: denaturation for 5min at 94°C, followed by 30 cycles of 30s at 94°C, 35s at 50°C, 1min 45s at 72°C, a final extension at 72°C for 10min. All products were verified on a 1% agarose gel and purified using exonuclease I (Exo I) and shrimp alkaline phosphatase (SAP) [36]. Purified DNA was sequenced by Macrogen Inc. (Amsterdam, Netherlands) on a 3730 DNA Analyzer.

In fish, the same mtDNA locus (*cox I*) was selected for a comparison of genetic diversity with the parasites. Primers for amplification of partial *cox I* sequence (~800 bp), FishF2 and VF2_t1, were adopted from [37]. The PCR mixture contained 12.5 μl of the PPP Master mix, 10.5 μl of ultra pure water, 5 pM of each PCR primer and 0.5 μl of isolated DNA (100 to 170ng). Cycling conditions were as follows: denaturation for 2 min at 94°C, followed by 35 cycles of 30s 94°C, 30s at 54°C, 1min at 72°C, and completed by 10min at 72°C (see 37). All products were verified on an agarose gel, purified and sequenced using PCR primers as in *Wenyonia*.

### Amplified fragment length polymorphism

Restriction and ligation of genomic DNA was performed using AFLP Core Reagent Kit (Invitrogen) and following the manufacturer’s manual. To reduce the number of fragments in the mixture, ligated DNA fragments were amplified by PCR (Pre-selective PCR) using primers complementary to the adapter and restriction site sequence extended with an additional selective nucleotide at their 3'—ends (EcoRI-A and MseI-C) [38]. The PCR mixture consisted of 2 μl of restriction-ligation product, 2.5 μl 5x MangoTaq^™^ Coloured Reaction Buffer, 0.75 μl MgCl_2_ (50mM), 0.625 μl of EcoRI-A and MseI-C primers (10pmol), 0.1 μl BSA (1mg/μl), 1.25 μl dNTPs (8mM), 4.6 μl ultra pure water and 0.05 μl MangoTaq^™^ DNA Polymerase (Bioline) (5U/μl). Cycling conditions for preselective-PCR were as follows: denaturation for 1min at 95°C, followed by 45 cycles of 15s at 95°C, 15s at 55°C, 30s at 72°C; and completed by 7min at 72°C. Fragments were verified on a 1% High Resolution Agarose gel (Invitrogen). This step was followed by selective amplification with primers containing 3 selective nucleotides at their 3'—end. Forward primers were labeled with different fluorescent colours on their 5'—end (EcoRI-ANN* and MseI-CNN) [38] to allow multiplexing in fragment analysis. Selective PCR consisted of 2μl of Pre-selective PCR product diluted by ultra pure water 1:10, 2.5 μl of 5x MangoTaq Buffer—coloured, 0.5 μl MgCl_2_ (50mM), 0.05 μl of EcoRI-ANN* fluorescent labeled primer (10pmol), 0.07 μl of MseI-CNN (10pmol), 0.1 μl of BSA (1mg/μl), 0.65 μl of dNTPs (8mM), 6.52 μl of MQ water and 0.1 μl of MangoTaq DNA Polymerase (Bioline) (5U/μl). Cycling conditions for selective PCR were as follows: denaturation for 2min 94°C, followed by 10 cycles of 20s at 94°C, 30s at 66°C (decreasing by 1°C every cycle for 10 cycles), 2min at 72°C; then 20 cycles of 20s 94°C, 30s at 55°C, 2min at 72°C and completed by 15min at 60°C. Amplified fragments were checked on 1% High Resolution Agarose gel (Invitrogen) and analyzed on ABI3730XL sequencer by Macrogen Inc. (Seoul, South Korea). Primer combinations (Table 2) were chosen according to available literature and published results for both *Wenyonia* [39–42] and for *Synodontis* [12,43,44].

**Table 2:** Primer combinations used for AFLP.

*Synodontis* spp.		*Wenyonia* spp.	
EcoRI-ACA*	MseI-CTC	EcoRI-ACA*	MseI-CTC
EcoRI-ACT*	MseI-CTA	EcoRI-ACT*	MseI-CTA
EcoRI-ACG*	MseI-CAA	EcoRI-ACG*	MseI-CAA
-	-	-	MseI-CAG
-	-	-	MseI-CAC

Asterisk indicates fluorescent-labelled primers.

### Phylogenetic analysis

Sequences were assembled, inspected for errors and aligned using GENEIOUS Pro software package version 6.1, software MAFFT [45,46]. Due to low variability of fish sequences, genetic distances in the host dataset varied between 0 and 1.5%. Hence, phylogenetic trees were reconstructed only for the parasite dataset. Suitable models of molecular evolution were selected using jMODELTEST 0.1.1. [47]. The model with the best likelihood was chosen using AIC criteria and phylogenetic trees were reconstructed using Bayesian inference (BI) in the program MrBayes v. 3.2.2. [48,49]. MrBAYES analyses were run for 15 million MCMC generations, with four chains and three independent runs and were performed under the HKY+G model of molecular evolution. Convergence was checked in AWTY [50]. Maximum-likelihood (ML) phylogeny was generated using the PhyML 3.0 software [51] under the HKY+G search parameters [52]. Initial trees were generated by an improved version of the neighbor-joining algorithm (BIONJ) [53]. Reliability of branching patterns within trees was tested by the bootstrap method with 1000 resamplings

As suggested for the coding genes (e.g. [54]), we assessed best-fit partitioning schemes for cox I using PartitionFinder [55]. Optimal partitioning schemes were set for MrBayes to perform BI runs and for all available models to run ML topologies. The best models were selected according to the AIC criteria as specified in Table 3.

**Table 3:** Substitution models for nucleotide data partitions of the *Wenyonia* spp. coxI dataset selected using the AIC in PartitionFinder for BI and ML runs.

Codon position	MrBAYES models	All models
1^st^	F81+I+G	F81+I+G
2^nd^	GTR+G	TrN+G
3^rd^	GTR+I+G	TrN+I+G

BI analyses were performed by software MrBAYES, run for 20 million MCMC generations, with four chains, three independent runs and were performed under the selected partition models; the coherence of each run was checked using software TRACER v1.5 [56]. ML analyses were carried out in the program GARLI 2.0 [57] under the selected substitution models and with automatic termination conditions.

All phylogenetic topologies (BI and ML) of *Wenyonia* dataset were rooted using sequences of *W*. *minuta*, representing the closest available sequence from Caryophyllidae.

### Haplotype networks

To examine the evolutionary relationships among haplotypes in populations of each parasite and host species statistical parsimony networks based on pairwise differences were constructed using PopART v1.7 [58].

### Population genetics statistics - mtDNA

To characterize the diversity of populations and their demographic history we performed several population genetic statistics for parasite and host mitochondrial datasets. Statistics of nucleotide genetic diversity (*Pi*), Haplotype diversity (*Hd*), Tajima’s test (*D*) and Fu & Li’s test (*D*) were calculated using DNASP v5 [59]. The statistical significance of *D* values were tested by 10,000 coalescent simulations [59].

### Gene flow and migration

**Fig 2:**
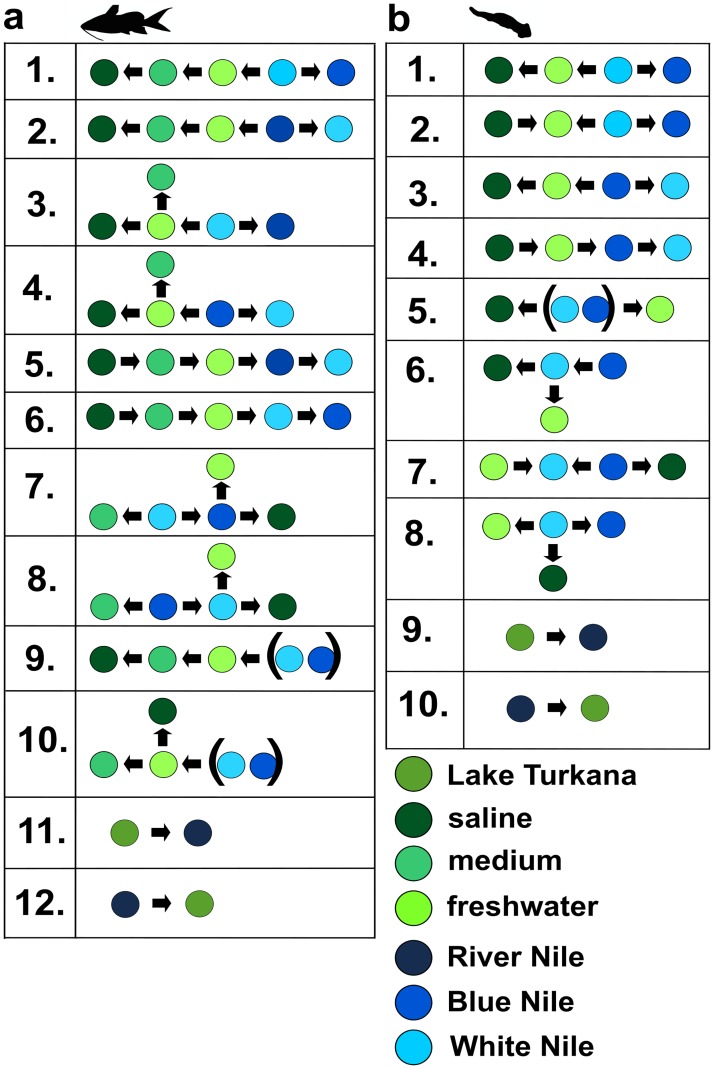
Migration hypotheses tested by software MIGRATE, datasets represented here are a) host; b) parasite. Arrows depict direction of historical gene flow.

The direction of historical gene flow was explored using coalescent based software MIGRATE v3.2.16 [60]. Different pathways of migration and colonization were tested to identify the ancestral population and to assess the direction of gene flow. Twelve and ten alternative migration hypotheses were tested for the host and parasite dataset, respectively (Fig 2a and 2b). Although representing similar scenarios, the hypotheses for hosts and parasites are not entirely identical. Only two parasite individuals were obtained from the medium salinity area in Lake Turkana (Kalokol) and thus this locality was excluded from the analysis of the parasite dataset. In the host dataset, hypotheses 1–10 represent scenarios of gene flow between individual localities, whereas hypotheses 11 and 12 represent general patterns of gene flow between Lake Turkana and the Nile. The same approach was adopted for the *W*. *virilis* dataset (see Fig 2b). Mitochondrial datasets were analyzed under HKY+G Model in the Bayesian mode of the program. Migrate runs were initiated using the following values of θ (theta) (minimum 0.0, maximum 1.0 and δ = 0.1) and M (migration) (minimum 0.0, maximum 1000.0 and δ = 100). Heating was set for five Multiple Markov Chains (1.0, 1.5, 3.0, 10000.0, 100000.0) and a swapping interval between chains of 1. The performance of the MCMC processes was checked for coherence and sufficient effective sample sizes (ESS) in Tracer v1.5 [56]. According to the ESS values obtained from preliminary runs, 90 million and 200 million generations were selected for *Wenyonia* and *Synodontis* datasets final analyses. Marginal likelihoods of different migration hypotheses were compared, and the best performing hypothesis was selected using Bayes Factors [61,62].

### AFLP data analysis

Analysis of the AFLP fragment data was performed using the GeneMapper Software v3.7 (Applied Biosystems). The thresholds were set for each primer combination (S1 Table). Each threshold was set as a 5% value of the highest peak fluorescence intensity. Selected peaks were manually checked for presence of any ‘noise’. All peaks above the threshold limit were exported, obtaining 6038 and 2169 loci for the parasite and host datasets, respectively. The output was transformed to a binary format using MICROSOFT EXCEL 2011 and then converted to GenePop file format using GenAlEx v6.5 [63]. Selectively neutral alleles suitable for analysis of population structure were filtered from loci under putative selection using MCHEZA [64]. The initial analysis was performed with 100 000 simulations and with default settings for confidence intervals, Θ, β-a, β-b and critical frequency. Candidates included for balancing and positive selection were removed from the dataset and the same simulation was run again. This procedure resulted in 716 loci for the *Wenyonia* dataset and 532 loci for *Synodontis* dataset. Principal Coordinate Analysis (PCoA), which was based on Nei’s genetic distances of markers, was computed in GenAlEx to visualize any emerging patterns of genetic structure in populations. To quantify the level of genetic variation between populations explained by geography were carried out in GenAlEx using AMOVA. Calculations were performed for host and parasite data from the Turkana freshwater and saline localities. The significance of the estimations was obtained with 999 permutations of the data. To estimate the number of population clusters or groups (K) in the dataset a Bayesian analysis was carried out using the software STRUCTURE v2.3.3 [65]. STRUCTURE was run using 15 independent calculations for each K, with values of K from one to ten for *Wenyonia* and from one to nine for *Synodontis*. The number of MCMC generations and burn in for both datasets was 3 million and 300,000, respectively. Runs were performed for both models of population history (admixture and noadmixture). Mean value of posterior probabilities, L(K), was determined for each K value. Since L(K) did not reveal a sharp peak, the rate of change between posterior probabilities of successive runs (delta K statistics, [66]) was calculated to allow estimation of the optimal value of K. Multiple runs generated by STRUCTURE were analyzed using the cluster matching and permutation program CLUMPP v1.1.2 [67] and graphically displayed as bar plots in Distruct [68].

## Results

A total of 347 sequences (990 bp) representing 209 haplotypes were obtained from *Wenyonia* spp. as follows: *W*. *virlis*—148 samples from the Turkana-freshwater site, 102 from the Turkana-saline site, 50 from the White Nile, 7 samples from the Blue Nile, 1 sample from the Atbarah River; 4 samples of *W*. *minuta*; 13 samples of *W*. *youdeoweii*. For *Synodontis* spp.,120 sequences (604 bp) representing 20 haplotypes were obtained: 32 samples from the Turkana-saline site (*W*. *virilis* prevalence 43.75%), 39 from the Turkana-freshwater site (prevalence 17.94%), 10 from the Turkana-medium site (prevalence 20%), 14 samples from the White Nile (prevalence 61.11%), 21 samples from the Blue Nile (prevalence 17.24%); 6 samples of *S*. *nigrita* (*Wenyonia* absent).

### Phylogenetic analysis

**Fig 3:**
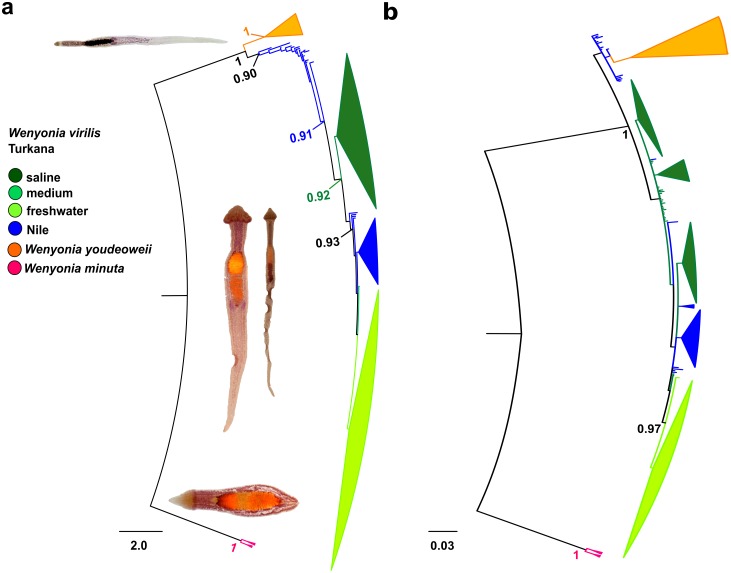
Phylogenetic tree of the *cox I Wenyonia* spp. computed by MrBayes for a) the model of molecular evolution; b) the best-fit partitioning schemes. Branches are collapsed into groups, which correspond to sampling locations. Statistical support for each group was obtained from the Bayesian posterior probability.

The results for analyses performed under the model of molecular substitution (MMS) and partition schemes (PS) differed in the position of *W*. *youdeoweii* in obtained phylogenies (Fig 3). In MMS, *W*. *youdeoweii* formed a clade separate from *W*. *virils* and *W*. *minuta*, whereas under PS settings, *W*. *youdeoweii* clustered within *W*. *virilis*.

Regardless of the differences in position of *W*. *youdeoweii*, the information obtained for *W*. *virilis* was similar in both analyses. Samples of *W*. *virilis* formed four groups. The resulting groups almost exclusively contained samples from either the Nile or the Lake Turkana. Neither of the basins formed mutually monophyletic clades, the Turkana groups were embedded in the Nilotic clades. The most basal *W*. *virilis* clades comprised exclusively samples from the Nile. The group derived from them was formed by individuals from the saline part of Lake Turkana. A second Nile cluster was located in the centre of the tree crown. The phylogenetically most derived group consists of samples from the freshwater part of Lake Turkana. This result supports the hypothesis that the Nile represents the ancestral population and the Turkana basin was subjected either to repeated colonization, or to bottlenecks reducing ancestral diversity of the lineages.

The unstable position of *W*. *youdeoweii* was also revealed by ML analyses (S1 Fig). In the ML analysis run under MMS, *W*. *youdeoweii* clustered inside *W*. *virilis* clade, while under PS, *W*. *youdeoweii* formed a separate clade sister to the *W*. *virilis* cluster. Regardless of the conflicting results in both types of analyses, the informative value of the ML results was less reliable due to very low bootstrap support.

### Haplotype networks

**Fig 4:**
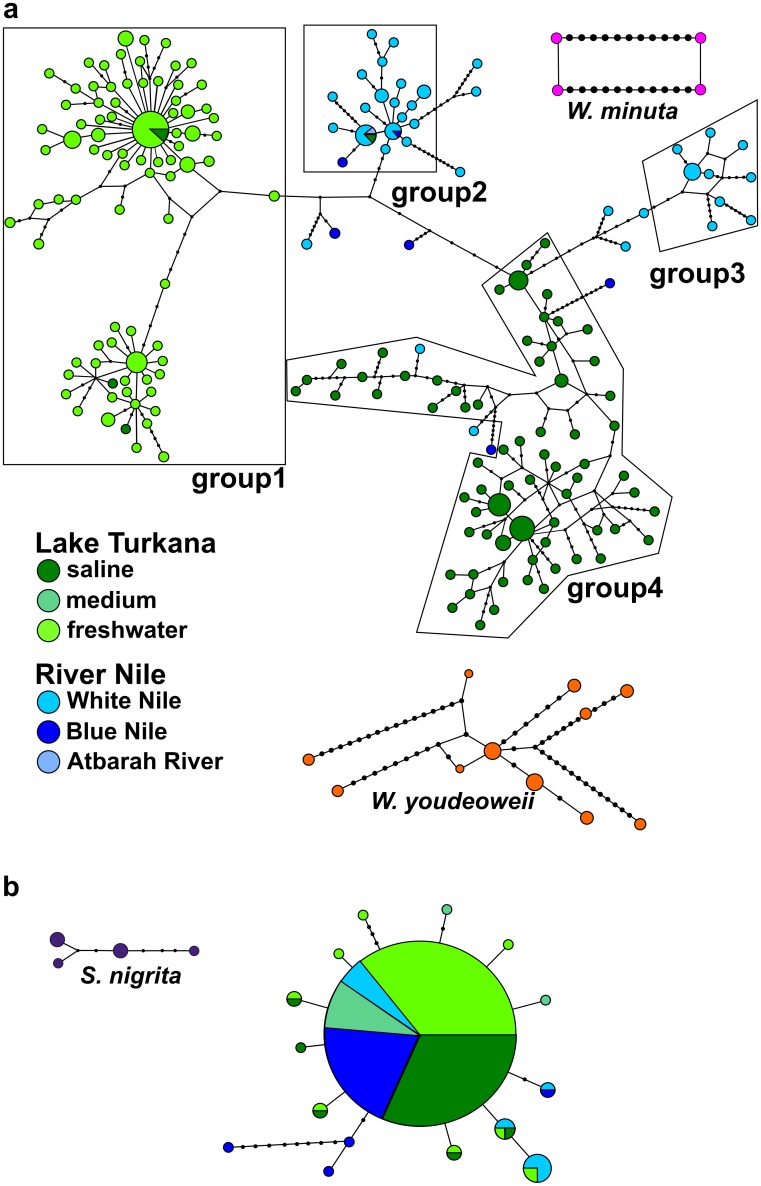
Haplotype network for the *cox I Wenyonia* spp. dataset (a) and for the *cox I Synodontis* spp. dataset (b) computed by PopART v1.7. Most groups were separated from each other by a maximum of six mutations. The exception was group 4, which was separated by ten mutations from group2 and eleven mutations from group3. Samples of *S*. *nigrita* clustered together and created a separate network. The sizes of haplotypic nodes are relative to the sample size.

Specimens of *W*. *minuta* and *W*. *youdeoweii* were clearly separated from those of *W*. *virilis* (Fig 4a). Apart from the remarkably high number of different haplotypes, i.e. the high ratio between the number of haplotypes and the number of individuals (194:347), the haplotype network revealed a considerable complexity of the haplotype grouping (Fig 4a). Individuals of *W*. *virilis* fell into four main groups. Samples from the freshwater Turkana locality exhibited a radial branching pattern with the main haplotype surrounded by satellite low frequency haplotypes—a trait typical for young expanding populations. In contrast, the populations from the Nile contained (with one exception) only haplotypes organized into networks separated by multiple mutations without a star-like patterns—a pattern typical for older populations in equilibrium (Fig 4a groups 2 and 3). Interestingly, groups 1 and 4 from Lake Turkana showed strong affinity either to the freshwater, or the saline part of the lake. Moreover, one Lake Turkana freshwater haplotype also occurred in the saline part of the lake, but not vice versa. Samples from the Nile fell into two groups (2 and 3), but four individuals were also placed in group 4 otherwise containing only samples from the saline part of Lake Turkana.

A total of 120 sequences representing 20 haplotypes of *Synodontis* spp. were used to create the haplotype network (Fig 4b). Six individuals of *S*. *nigrita* produced a separate cluster with four haplotypes, whilst the rest of the 114 sequences of *S*. *schall* from the Nile and Lake Turkana clustered together. The two networks were separated by 55 mutations. In contrast to the genetically diverse parasite, the host network was distinctly less variable. The central *Synodontis* haplotype in which the majority of specimens clustered was represented at all localities in both basins, only two haplotypes that were separate from the central haplotype by more than two mutations were found (Fig 4b).

### Population genetic analysis of mtDNA sequences

The differences between the host and parasite populations seen in their haplotype networks were also reflected in their population genetic statistics (Table 4). Results of the DNA polymorphism statistics for *W*. *virilis* showed high values of haplotype and nucleotide diversity and small differences between the Turkana and Nile basin. In *Synodontis*, values of all statistics were considerably lower for Turkana populations and both Tajima’s and Fu and Li’s tests showed significant negative values. The results indicate that the Turkana populations are genetically smaller and expanding, which is typical for populations subjected to a recent bottleneck.

**Table 4:** Results of population genetic statistics of mtDNA for both parasites and hosts.

	*Wenyonia virilis*		*Synodontis* spp.	
	Nile	Turkana	Nile	Turkana
Haplotype diversity	Hd = 0.98	Hd = 0.986	Hd = 0.631	Hd = 0.389
Nucleotide diversity	π = 0.016	π = 0.015	π = 0.004	π = 0.001
Tajima’s D	D = -1.211*	D = -1.271*	D = -1.37	D = -2.40*
Fu and Li’s D	D = -3.123	D = -4.56	D = -1.73	D = -4.00*

Asterisk indicates values significant at P<0.05.

### Gene flow and migration

The likelihood scores and probabilities of the hypotheses tested using MIGRATE software are shown in Table 5. Despite the fact that one of the analyses of the *Synodontis* dataset favoured the hypotheses assuming a gene flow from Lake Turkana (hypothesis 11), an analysis of with the dataset split into individual localities revealed that the Lake Turkana population originated in the White Nile (hypothesis 3, Table 5). In contrast, for *Wenyonia* spp. the direction of gene flow was the same for both analyses. The results supported the hypothesis of gene flow from the Nile to Lake Turkana (hypothesis 10) and independent colonization of the Blue Nile as well as of the freshwater and saline areas of Lake Turkana from the White Nile (hypothesis 8). Similarly, according to the gene flow and migration analyses of *Synodontis*, the ancestral population was located in White Nile from where the colonization wave spread to the Blue Nile and Lake Turkana (hypothesis 3). Within Lake Turkana, the freshwater area was colonized first, while saline and medium salinity areas were populated separately. However, this colonization sequence is not completely testable for parasites due to their low prevalence in the medium salinity part of Lake Turkana (host n = 23, only 2 infected).

**Table 5:** Results of the *Synodontis* spp. and *Wenyonia* spp. migration hypotheses tested with the coalescent based software MIGRATE, showing comparisons of marginal likelihoods and model probabilities for each dataset.

	*Synodontis* spp.		*Wenyonia* spp.	
	Marginal LH	Model Probability	Marginal LH	Model Probability
H1	-2098.6	1.55 e^-222^	-26193.9	7.36 e^-277^
H2	-2551.8	0	-27100.8	0
H3	**-1843.2**	**1**	-32718.3	0
H4	-2455.6	0	-26171.8	1.26 e^-257^
H5	-1878.0	5.61 e^-31^	-27224.7	0
H6	-2178.9	2.71 e^-292^	-26527.2	0
H7	-1879.7	1.94 e^-32^	-26254.0	0
H8	-2003.1	1.30 e^-139^	**-25876.0**	**1**
H9	-2201.9	0	-28132.6	0
H10	-2180.9	4.33 e^-294^	**-26607.9**	**1**
H11	**-1536.4**	**1**	-	-
H12	-1990.3	0	**-**	**-**

The best-supported hypotheses are in bold; H1-Hn refer to corresponding hypothesis {Marginal Likelihoods were used to calculate the Bayes factors (model probability) and for comparing alternative models of gene flow}.

### Population genetic structure—AFLP

**Fig 5:**
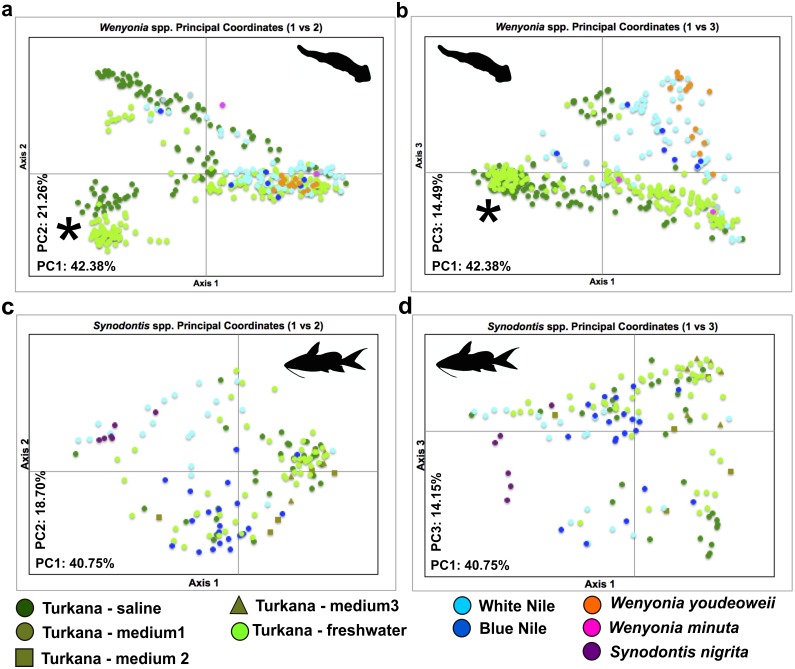
Results of PCoA for (a, b) *Wenyonia* spp. and (c, d) *Synodontis* spp. based on the genetic distances generated by GenAlEx software. Results are presented for the first three axes, because the second and the third axis explained similar levels of variability. In PCoA, all individuals were labelled with different colours based on their geographical or species origin. Asterisks (a, b) indicate clearly separated clusters comprising solely *Wenyonia* Turkana samples. *Synodontis* samples (c, d) from all localities clustered together without forming distinguishable groups. *S*. *nigrita* individuals clustered together and even showed a tendency to create a separate group (d). Turkana—medium 1–3 refers to three different localities: 1, Central Island; 2, Kerio River delta; 3, Kalokol.

AFLP data were obtained for 368 parasites using 15 primer combinations and for 138 hosts using 9 primer combinations (Table 2). The final dataset contained 716 and 532 fragments for parasite and host, respectively. The analysis performed for parasites (Fig 5a and 5b) produced a pattern indicating possible formation of multiple clusters. Surprisingly, all three tapeworm species clustered together, with an apparent tendency towards segregation of most of the individuals from the saline part of Lake Turkana and also for separation of Lake Turkana from the Nile. A separate, smaller cluster containing exclusively Turkana samples was clearly subdivided into two groups, according to their origin either in the freshwater, or saline sites. Note that the larger cluster comprises samples from all localities, but also contains *W*. *youdeoweii* and *W*. *minuta* samples, which did not create separate units (compare with mtDNA analysis Figs 3 and 4). The smaller cluster contained exclusively samples from Lake Turkana (bottom left). The cluster is clearly subdivided into two groups, according to their origin either from the freshwater, or saline part of the lake. When axis 1 and axis 3 were compared, the majority of the samples from Lake Turkana formed a separated group (Fig 5b—bottom half).

**Fig 6:**
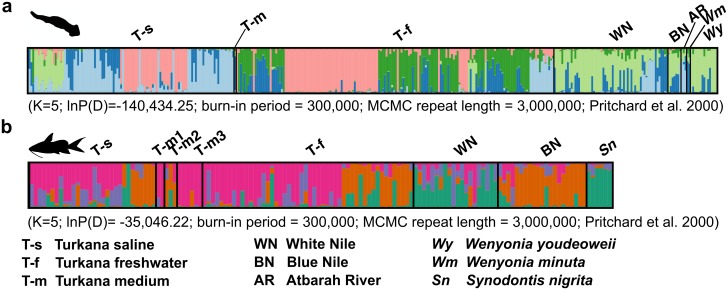
Individual-based cluster representation of all sampled *Wenyonia* spp. (a) and *Synodontis* spp. (b) as revealed by the Bayesian inference of population structure. Each colour represents one assumed population cluster K. Multiple coloured bars display an individual’s estimated membership proportion in more than one population (q), i.e. admixture. Turkana—medium 1–3 refers to three different localities: 1, Central Island; 2, Kerio River delta; 3, Kalokol.

Results of the AFLPs PCoA analysis are different from the picture of the population structure obtained from mtDNA. PCoA clustering of individuals did not corroborate the separation of *W*. *virilis* specimens into mtDNA haplogroups, but showed some differentiation between the Turkana and Nile populations (Fig 5a and 5b). Moreover, AFLP markers did not separate *W*. *youdeoweii* and *W*. *minuta* from *W*. *virilis* in any of the performed analyses (Figs 5 and 6a). The AMOVA analyses based on *F*_*ST*_ calculation (Infinite Alleles Model) revealed that the majority of variation occurred within the sample sites for all datasets (90% for parasite, 93% for host in the whole dataset; 89% for the parasite, 98% for the host in Lake Turkana dataset). Among sites the population structure (Turkana vs. Nile) explained only 10% of variance for *Wenyonia virilis* and 7% of variation for *Synodontis* spp. Among sites the population structure between the Turkana freshwater and saline part explained 11% variation for *Wenyonia* virilis and 2% for *Synodontis* spp. (Table 6).

**Table 6:** Results of the AMOVA based on *F*_*ST*_ for *Wenyonia virilis* and *Synodontis* spp. AFLP data from Lake Turkana and the Nile; Turkana freshwater and saline part. Estimations are based on 999 permutations and the significant at P<0.001.

Source of varition	*df*	Sum of squares	Mean squares	Estimated variance	Explained variance (%)
**Turkana vs Nile**					
***Wenyonia virilis***					
**Among sample sites**	1	1559.575	1559.575	12.753	10%
**Within sample sites**	349	40106.447	114.918	114.918	90%
**Total**	350	41666.023		127.672	100%
**Synodontis spp**.					
**Among sample sites**	1	374.602	374.602	5.301	7%
**Within sample sites**	130	9738.262	74.910	74.910	93%
**Total**	131	10112.864		80.211	100%
**Turkana freshwater part vs saline part**					
***Wenyonia virilis***					
**Among sample sites**	1	1902.670	1902.670	13.434	11%
**Within sample sites**	278	30110.370	108.311	108.311	89%
**Total**	279	32013.039		121.744	100%
**Synodontis spp**.					
**Among sample sites**	1	132.709	132.709	1.551	2%
**Within sample sites**	78	5813.353	74.530	74.530	98%
**Total**	79	5946.063		76.082	100%

The PCoA for the host dataset (Fig 5c and 5d) revealed a similar pattern to the haplotype network of *cox I* sequences. The lack of population structure observed in AFLP corresponds with mtDNA data.

Population structure was further analyzed using the STRUCTURE software. The optimal number of genetic clusters (K) was estimated by using the delta K statistics (DK). The number of clusters in the parasite dataset was K = 5 for the admixture model of population history (Fig 6a) and K = 4 for the non-admixture model of population history (see S2 and S3 Figs). As in the PCoA analysis, *W*. *youdeoweii* and *W*. *minuta* were not separated from *W*. *virilis* and shared clusters with samples from the Nile (Fig 6a). The Nile population was characterized by the predominant occurrence of one cluster (lime green), supplemented with two other clusters (light and dark blue). Samples from Lake Turkana shared clusters with the Nile (light blue, dark blue, less frequently lime green), but a unique Turkana cluster was present with high frequency (magenta). Samples from the freshwater part also frequently contained another dark green cluster, which was almost absent in Nile samples. The population from the saline area also contains a unique cluster (magenta). Clusters shared between the saline population and the Nile are different from the freshwater part of Lake Turkana (light blue, lime green, dark blue).

Clusters, which are less frequent in the Nile (light blue, dark green), showed an increased proportional presence in Lake Turkana, concordant with the hypothesis of the Nilotic origin of the Turkana populations. The presence of different Nile clusters in the freshwater and saline parts of Lake Turkana might reflect multiple colonization events. Similar to the PCoA, AFLP clusters obtained in STRUCTURE did not match with specific mtDNA haplogroups. *Wenyonia youdeoweii* and *W*. *minuta* did not separate from *W*. *virilis*.

In the host dataset, results distinguishing the four clusters (admixture population model; K = 5 for no-admixture historical model S3 Fig) received the highest peak in the DK statistics (S2 Fig). All four clusters were present at all localities from the Nile and Lake Turkana albeit with different frequencies (Fig 6b). The cluster with the highest frequency in Lake Turkana (pink) has the lowest frequency in the Nile, whereas the main clusters in the Nile (green and purple) were less frequently observed in Lake Turkana, a pattern similar to that revealed in the parasite. Interestingly, the two Nile branches, Blue and White Nile, differed in the composition of their clusters too. Contrary to the PCoA analysis, *S*. *nigrita* did not show a tendency to cluster separately and shared clusters with *Synodontis* samples from the White Nile.

## Discussion

Two different markers, AFLP and mtDNA, were used to assess the levels of genetic diversity, population structure and shared evolutionary patterns in a host-parasite system in two recently divided basins. Evidence of geographically determined events shared between the two organisms was revealed in the form of a historical bottleneck and Nilotic origin of the Lake Turkana populations. Additionally, an indirect impact of salinity gradient on the structure of Lake Turkana parasite populations was indicated.

### Fast evolving parasites vs. stationary hosts

The markedly faster rate of molecular evolution reflected by an increased sequence diversity of *W*. *virilis*, when compared to its host possessing similar generation time (one year), is striking. We were not able to provide an exact calculation of the difference in the mutation rates due to the character of our data. Methods used for such estimations rely on clade to clade comparisons (e.g. *BEAST [69]), whereas our fish samples lacked any such structure. Despite that, it is clear that the mutation rates must differ dramatically (sequence variability for *Wenyonia virilis* dataset is between 100–95.5% vs. 100–97.3% for *Synodontis*). An increased mutation rate in parasites is a common feature [70,71] and a shorter generation time of the parasite is the most common explanation for the discrepancy [72]. However, in our case, organisms with similar generation times were studied. Instead, several other evolutionary factors might be involved. For example, it was suggested that higher migration rates of the host could increase mutation rate in the maladapted populations of the parasite [73,74]. However, multiple factors, such as differences in effective population sizes, species-specific historical events, or a higher life expectancy of the host, may also play a role. Although frequently referred to in the literature [75–77], the phenomenon of the slowly evolving hosts opposed by rapid evolution in their parasites remained surprisingly poorly documented until recently. To our knowledge, the *Wenyonia-Synodontis* model represents the only example documented in metazoan endoparasites, as most other examples include exclusively ectoparasites and Apicomplexa [70,72,78,79]. Hence, when supplemented with more genes and species, our system might provide an alternative model for the studies of host-parasite coevolution.

### Opposing population histories of host and its parasite

Both mtDNA analyses showed that tapeworms from the Nile formed two separate groups (Figs 3 and 4a). Each of them was phylogenetically more closely related to one of the two Lake Turkana (sister) groups than to each other. Interestingly, each of the two Lake Turkana groups was almost exclusively restricted either to the freshwater or the saline part of the lake (Fig 4a). This pattern might indicate multiple colonization waves of Lake Turkana from the Nile and the individual haplogroups might be considered to be result of different colonization events. Moreover, the apparent absence of gene flow between the saline and freshwater Turkana haplogroups implies lineage fission. Alternatively, the haplogroups could be interpreted as remnants of the initial ancestral polymorphism introduced during colonization, which was later reduced through genetic drift or selection.

A similar pattern to AFLP data was seen in the PCoA results. Despite poor population structure resolution the clear separation of a cluster comprising solely Lake Turkana samples (Fig 5a and 5b asterisk) from a cluster containing samples from both basins provides further support for multiple migration events. Thus, both types of data—mitochondrial and total genomic—provide an indication of multiple migration events. This correlates with the well-documented Holocene re-connections of the Nile and Turkana basins, and provides a plausible explanation for the occurrence of multiple, genetically differentiated haplogroups in each basin (Fig 4a).

The AFLP data were structured into 5 clusters; only one of them (magenta) was unique and occurred in both the saline and the freshwater part of Lake Turkana. The abundance of the unique Turkana cluster (Fig 6a) was not as high in the saline part as it is in the freshwater area. This could indicate that saline and freshwater environments might have different effects on populations, e.g. by means of different osmotic conditions or differences in the spectrum of available intermediate annelid hosts.

In contrast to the parasite, the multiple colonization events in Lake Turkana coupled with the marked similarity of the Nile and Turkana *Synodontis* populations revealed by both mtDNA and AFLP data might be well explained by genomic admixture among multiple founding populations, i.e. lineage fusion. A phenomenon characteristic for some taxa in re-colonized insular biogeographic systems, such as in giant tortoises *Chelonoidis becki* from Galápagos Islands [80,81]. Thus, in our model, the lineage fusion pattern in the host is virtually opposite to the lineage fission, i.e. ongoing subpopulation segregation in the parasite.

### Signs of parasite’s allopatric differentiation in a sympatric system?

Following the end of the Pleistocene megadrought hypersaline conditions, the Turkana basin underwent severe environmental oscillations. During the Holocene freshwater periods, *Wenyonia virilis* populations were most probably widespread, whereas under current conditions they are restricted to areas where suitable, presumably freshwater, intermediate hosts are available. This assumption is strongly supported by the near absence of *Wenyonia* spp. in the medium salinity part of Lake Turkana that lacks permanent freshwater habitats. The presence of freshwater springs in the saline part of Lake Turkana [18], together with the common occurrence of *Wenyonia* tapeworms, confirm that at least locally a suitable (presumably freshwater) intermediate host is also present in the saline part of the lake. As there are no physical obstacles to host migration in Lake Turkana, isolation by adaptation can better explain the extent of the tapeworm diversification rather than isolation by distance [82–84]. The restricted distribution and the lack of substantial gene flow between the ‘freshwater’ and ‘saline’ *Wenyonia* (haplo) groups might be caused by their different environmental requirements, possibly including the role of the intermediate host(s).

The revealed restriction of the ‘freshwater’ subpopulation to a freshwater refugium of the Omo delta (and perhaps lower Omo) could be explained by highly saline conditions throughout most of the open lake. However, restriction of the ‘saline’ *Wenyonia* population to a particular part of the lake, makes adaptation of the two *W*. *virilis* subpopulations to the saline or freshwater conditions *per se* unlikely.

The documented, but quantitatively slight, geographical overlap between freshwater and saline haplogroups, i.e. sporadic occurrence of tapeworms of the freshwater haplogroup in the saline part of the lake, might be explained by migration of hosts within the lake [27]. This interpretation is supported by two different phenomena observed in our model: i) the apparent lack of actual gene flow between freshwater and saline haplogroups, and ii) the complete absence of mixed infections based on a robust dataset comprising 347 tapeworms and 120 hosts analyzed where all individual hosts were infected by tapeworms belonging either to the freshwater or saline haplogroup only. Importantly, common co-infections with up to three *Wenyonia* spp. in an individual *Synodontis* hosts in both basins [31] make competitive exclusion unlikely to be responsible for this pattern.

In fact, the pattern observed in the Lake Turkana parasite population might reflect an uneven or non-overlapping distribution of different intermediate host species, to which individual tapeworm subpopulations might have adapted.

Hence, our data suggest that the salinity causes, though possibly indirectly, the apparent ecological and near-complete geographical segregation of parasite subpopulations. This might indicate that within a single (isolated) limnic system, salinity might facilitate sympatric speciation by means of ecological segregation of subpopulations triggered by the presence of physiochemically contrasting freshwater and saline environments. Analogical processes leading to the emergence of a salinity gradient affected many African basins, and thus could have a fundamental, yet overlooked, influence on the evolution of limnic organisms throughout the Sub-Saharan Africa and elsewhere.

The indication of multiple, at least two, colonization events has been shown herein by both markers. Two migration events are reflected in our dataset by the occurrence of multiple mt haplotypes in individual localities. Importantly, AFLP data are showing on-going segregation in freshwater and saline parasite (sub)populations that were probably established prior to the last colonization. However, this separation is still incomplete and population patterns shared between the Nile and Lake Turkana indicate that the last ~11 ky were not long enough to completely segregate the tapeworm populations.

Our finding of almost exclusively ‘freshwater’ and ‘saline’ tapeworm subpopulations with no apparent gene flow between them represents the first described case of allopatric differentiation (lineage fission) in a clearly sympatric system in the case of endoparasites (documented in ectoparasites only [85,86]).

### mtDNA versus AFLP

Compared to previous studies, which successfully used AFLP for a variety of purposes including evolutionary studies of populations, drug sensitivity or detection of inbred individuals [39,87–90], AFLP did not provide an optimal level of resolution in our study. Using solely AFLP data, we were not fully convinced that we could clearly distinguish whether the effect of IBA is overpowering IBD (i.e. isolation by adaptation or isolation by distance) in differentiating Lake Turkana populations.

Moreover, individual *Wenyonia* and *Synodontis* species were clearly differentiated by mtDNA data. Contrary to our expectations, the AFLP data reflected the interspecific genetic differentiation obtained from mtDNA only to a very limited degree, distinguishing *S*. *nigrita* in the host dataset but failing to clearly separate any of the three *Wenyonia* species. Similar to outcomes documented in insects [91] and attributed to deep genetic divisions, in which species/subspecies separated on the basis of mtDNA data are not always reflected in the nuclear genome. To some degree, the lack of differentiation in multilocus samples could be caused by undersampling (e.g. *W*. *minuta* with only 2 specimens available). However, it is certainly not the case in *W*. *youdeoweii* for which a representative population sample was obtained. Thus, it remains unknown whether the AFLP differences found in the Lake Turkana (sub)populations are directly associated with particular loci that are important for the development of differentiation/segregation between ‘freshwater’ and ‘saline’ populations, or not. Therefore, although we cannot rule out that the observed changes in genetic diversity have little or nothing to do with segregation, it is possible that we in fact demonstrate early evolutionary changes associated with it.

Although the AFLP data showed similar patterns in both organisms, the picture of population structure obtained from these data was much less clear. In particular, an increased frequency of some clusters from the Nile was seen in Lake Turkana and vice versa and unlike mtDNA, markers did not reveal any corresponding geographical pattern among *Wenyonia* samples from Lake Turkana. Moreover, the signal of the ancestral gene flow contained in the mtDNA data was similar for both organisms and provided valuable information about historical changes in species distributions. Therefore, the suitability of AFLP data to elucidate one of the main questions of this study, i.e. significance of the salinity gradient itself, remains unanswered. Compared with other studies this study is based on a sufficiently high number of isolated loci (e.g. 716 fragments for parasite and 532 for host; vs. 229 and 987 AFLP loci for Nematodes [39,92]; 731 and 237 loci for plants [93,94]; 672 loci for *Plasmodium* spp. [95]). Despite the large number of fragments analysed in both hosts and parasites, co-dominant markers like microsatellites or SNP could potentially provide more informative data. The low informativeness might be caused by suboptimal performance of the primers used for selective PCR in our assay. For example, PCR performance of the selective primers with 3 additional bases could have been too strict and therefore many fragments, which are shared within a population or a lineage, may have been missed. As a result, datasets might proportionately contain a low number of shared and a high number of rare loci/fragments, making detection of genetic patterns identifying different populations difficult.

### Conclusions

Despite the common origin of the Lake Turkana populations in the Nile, the population genetic pattern observed in hosts implies the fusion of multiple colonisation waves, contrasting with the lineage fission (segregation) in its parasite. This pattern might be explained by ecological permeability of the specific environment in which the saline part of Turkana poses a barrier for a parasite dependent on freshwater intermediate host(s), while allowing free dispersal of the definitive fish host. The parasite populations exhibited markedly higher molecular evolutionary rates compared to the hosts, providing a rare example of the long-discussed theory of rapid evolution in parasites compared to their hosts. Although AFLP and mtDNA showed similar patterns in both host and its parasite, mtDNA provided a remarkably higher resolution of population structure, historical gene flow, evolutionary patterns, and inter- and intra-specific genetic differentiation.

## Supporting information

S1 Table Discordant population histories—Fish and tapeworms. Thresholds were individually set for each AFLP primer combinations.
(XLSX)

S1 Fig Discordant population histories—Fish and tapeworms. Phylogenetic tree of *cox I Wenyonia* spp. for a) the model of molecular evolution computed by PHYML; b) the best-fit partitioning schemes carried out in Garli. Branches are collapsed into groups, which correspond to sampling locations. Statistical support for each group was generated from Likelihood bootstrap proportions.
(TIFF)

S2 Fig Discordant population histories—Fish and tapeworms. ΔK results for historical models admixture of *Wenyonia* spp. (a) and *Synodontis* spp. (b) and noadmixture of parasite (c) and host (d).
(TIFF)

S3 Fig Discordant population histories—Fish and tapeworms. Individual-based cluster representation of all sampled *Wenyonia* spp. (a) and *Synodontis* spp. (b) as revealed by Bayesian inference of population structure. Each colour represents one assumed population cluster K. Multiple coloured bars display an individual’s estimated membership proportion in more than one population (q), i.e. noadmixture. Turkana—medium 1–3 refers to three different localities: 1, Central Island; 2, Kerio River delta; 3, Kalokol.
(TIFF)
