# Supplementary figures and images for: Discordant population histories of host and its parasite: A role for ecological permeability of extreme environment?
Source: PLoS One. 2017 Apr 10;12(4):e0175286. doi: 10.1371/journal.pone.0175286 (PMC5386267; doi:10.1371/journal.pone.0175286)

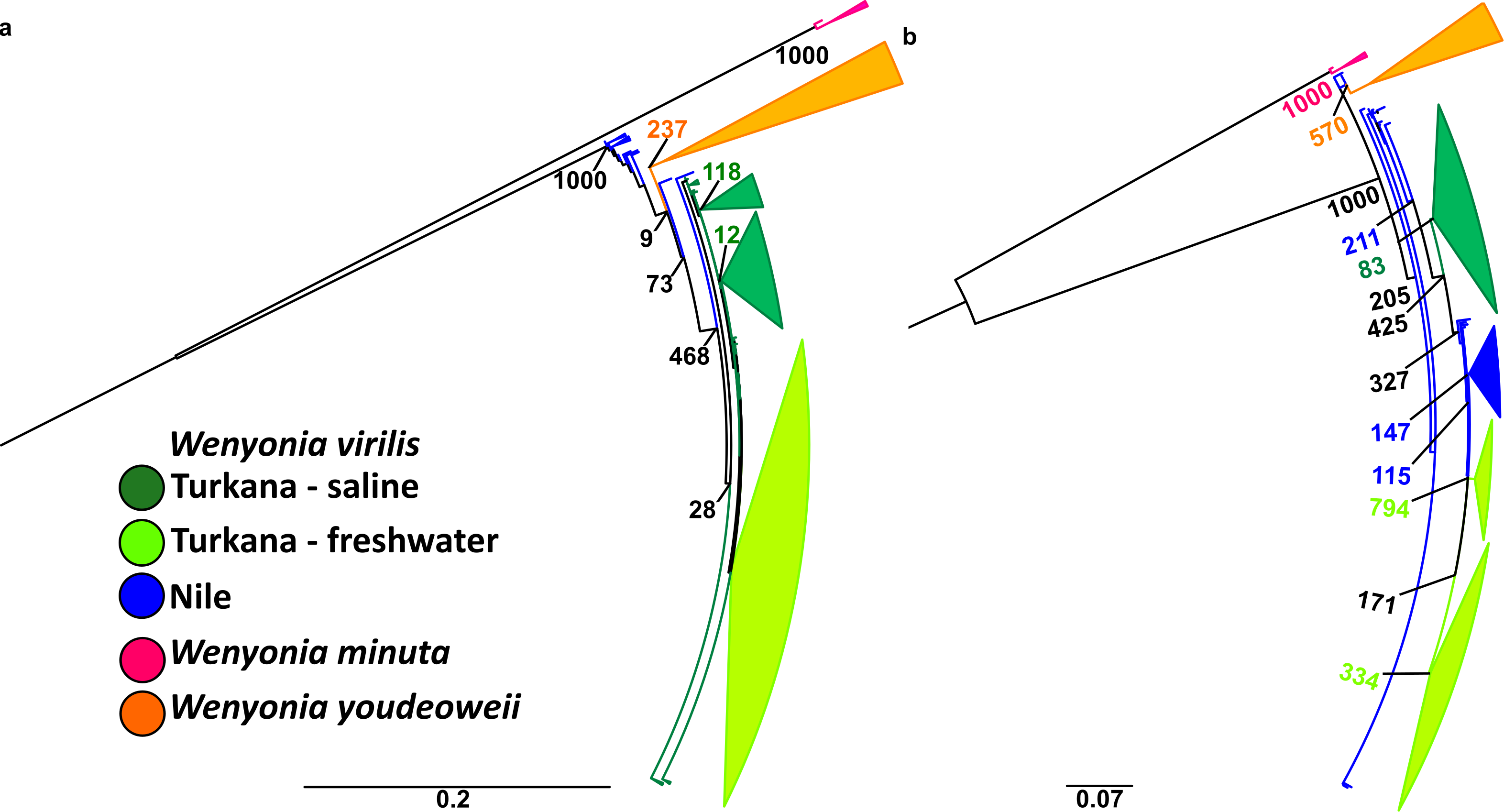

Supplement: S1 Fig — Phylogenetic tree of cox I Wenyonia spp. for a) the model of molecular evolution computed by PHYML; b) the best-fit partitioning schemes carried out in Garli. Branches are collapsed into groups, which correspond to sampling locations. Statistical support for each group was generated from Likelihood bootstrap proportions. (TIFF) [file pone.0175286.s002.tiff]

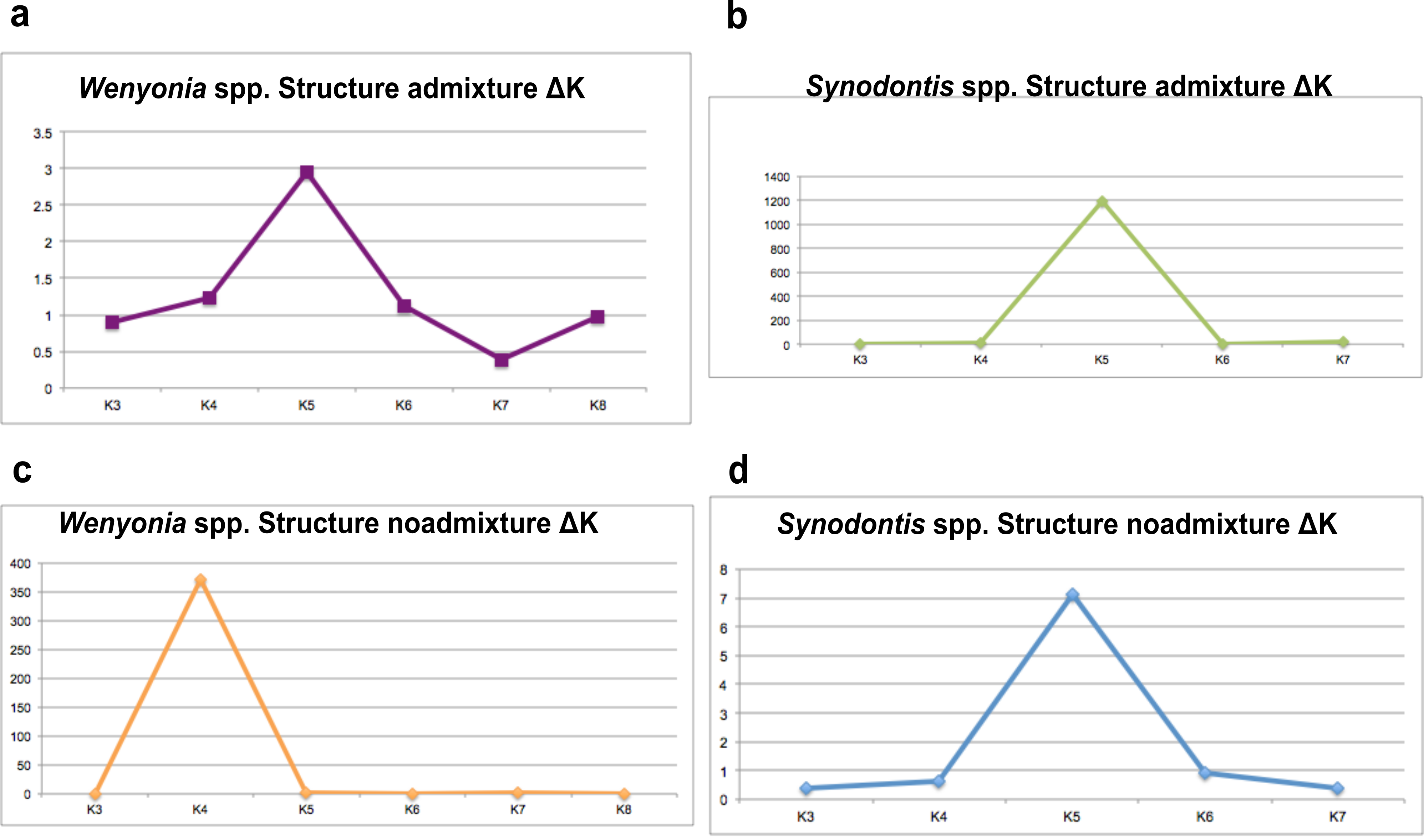

Supplement: S2 Fig — ΔK results for historical models admixture of Wenyonia spp. (a) and Synodontis spp. (b) and noadmixture of parasite (c) and host (d). (TIFF) [file pone.0175286.s003.tiff]

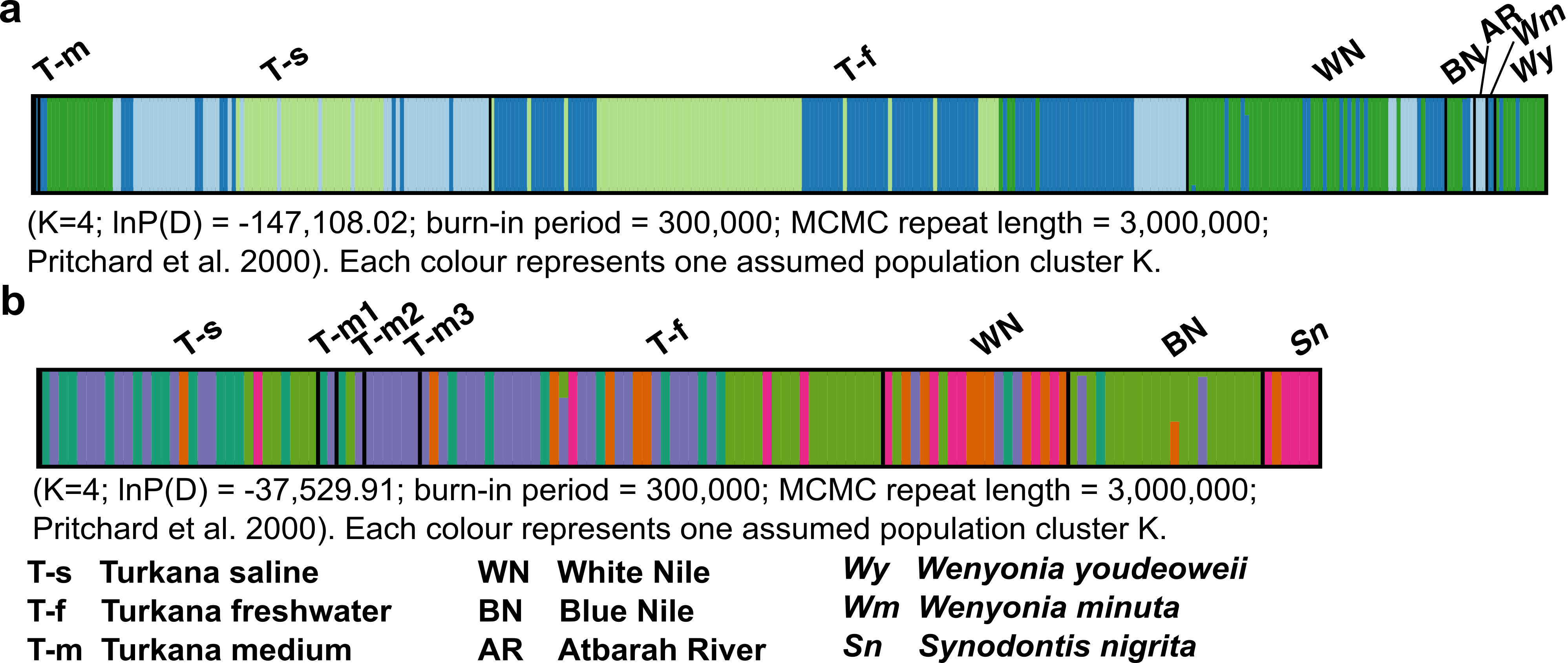

Supplement: S3 Fig — Individual-based cluster representation of all sampled Wenyonia spp. (a) and Synodontis spp. (b) as revealed by Bayesian inference of population structure. Each colour represents one assumed population cluster K. Multiple coloured bars display an individual’s estimated membership proportion in more than one population (q), i.e. noadmixture. Turkana—medium 1–3 refers to three different localities: 1, Central Island; 2, Kerio River delta; 3, Kalokol. (TIFF) [file pone.0175286.s004.tiff]
